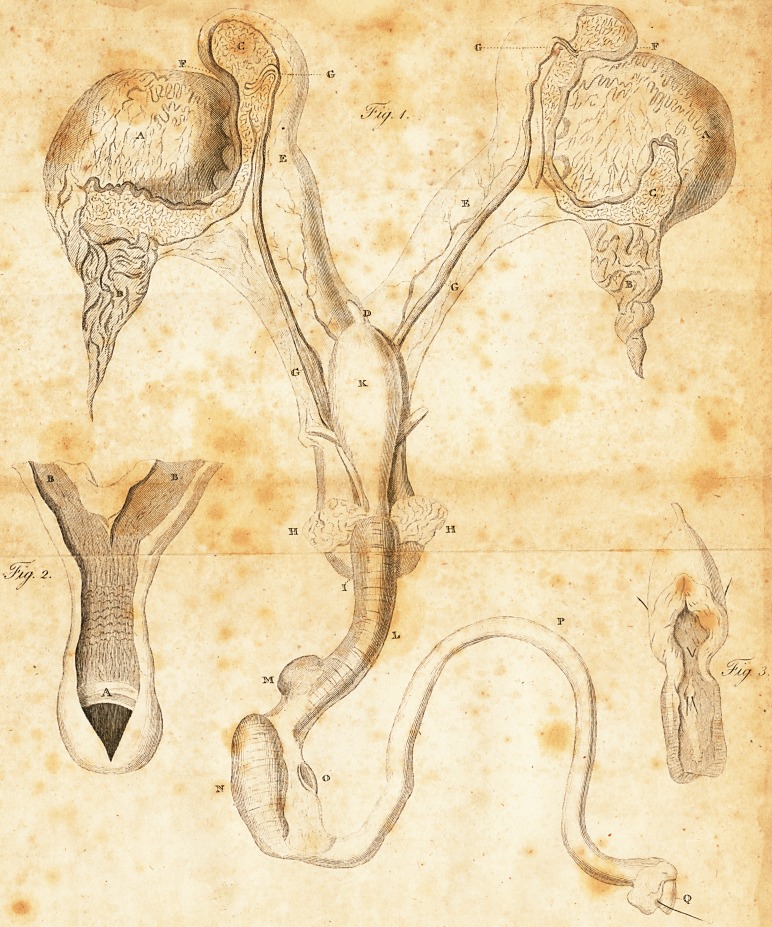# Mr. Thomas's Description of an Hermaphrodite Lamb

**Published:** 1799-08

**Authors:** H. Leigh Thomas

**Affiliations:** Leicester-Square


### Medical and Physical Journal.

VOL. II.] AUGUST, 1799. [NO. VI.

### *To the Editors of the Medical and Phyſical Journal.*

Gentlemen,

If the following deſcription of an hermaphrodite comes within the views of your uſeful publication, you have my permiſſion to inſert it whenever you think proper.

I remain, Gentlemen,

Your's very reſpectfully,

H. LEIGH THOMAS.

Leicester-Square,

*July* 21ſt, 1799.

Deviations from the natural ſtructure in animals do not frequently lead to any immediate improvements in the practice of medicine; but they tend to enlarge our knowledge of the animal economy, and may in this way be ſaid to contribute remotely to the advancement of the healing art.

The ſmaller deviations from the natural ſtructure in man, and in other animals, are very numerous; but even thoſe in the moſt important organs not unfrequently occur. That ſpecies of deviation or monſtroſity, called hermaphrodite, has hardly ever been known to happen in the human ſpecies; but, in ſome other animals, as in the bull and the ram, it has been occaſionally remarked.

The one which has lately occurred to me, and which I ſhall now attempt to deſcribe, is very complete in its kind, and is different in moſt reſpects from what has been publiſhed by Mr. Hunter, in his book, upon certain parts of the animal economy.

The lamb, the ſubject of the following deſcription, was not more than two months old; it had the external characters of a ram, as far as is common at that age, except a deficiency of the ſcrotum; having been purchaſed amongſt a number of others in a public market, no account of it previous to diſſection could be procured. Upon opening into the abdomen, the uterus appeared to occupy its uſual poſition, with reſpect to the urinary bladder and the rectum; the peritonæum was reflected over it in the uſual manner, forming its ligaments; the blood veſſels (which were afterwards filled with a coloured fluid) took the common route, and the uterus divided into two horns, which externally had the ufual appearance. The Fallopian tubes arofe out of them, and terminated in a tortuous, convoluted manner upon the body of a fubftance exactly refembling the tefticle of a ram. The body of the uterus poffeffed the common rugofe ftructure, but the horns were only lined by a fmooth membrane; thofe glandular bodies which are obfervable in the perfect uterus being altogether wanting. At the anterior extremity of the fundus uteri, a thick femi-lunar valve paffed acrofs, which feemed to correfpond to the os tincæ, and hardly allowed a fine probe to pafs over its upper edge. The vagina fcarcely exifted, forming only a fhort pouch beyond the valve, which was lined by a fmooth membrane, without any appearance of a fallicular ftructure.

The teftes occupied the place of the ovaria, and were inclofed by the fame reflection of the peritonæum which formed the broad ligaments of the uterus; they were of the common fize, and in form a little more globous than ufual, which perhaps may be explained from their never having been pendulous. The blood-veffels, after being filled with the red injection, appeared to take the ufual circuitous route, communicating with thofe of the uterus; and the lymphatics alfo feemed to arife, and pafs out by the fpermatic chord in the ufual order. A longitudinal fection being made through one of the tefticles, its internal ftructure was precifely the fame with what is natural to that organ; and upon maceration in water for a certain time, it put on the ufual fhaggy appearance formed by the tubuli femeniferi. The remafter mufcle was wanting, as well as the tunica vaginalis; the latter could not be obtained unlefs the tefticle had paffed the abdominal ring. The epididymis belonging to each tefticle prefented the common convoluted ftructure, and the canal was pervious throughout its whole length, quickfilver freely paffing along it from the vas deferens to the tefticle. The vas deferens, after leaving the epididymis, paffed down upon the outfide of the floras of the uterus, between the duplication of the peritonæum, and opened on each fide of the caput gallinaginis; the veficulæ feminales lay upon the fhort pouch correfponding to the vagina, ftill preferving their relative fituation with refpect to the urethra; they were of the common fize and ftructure, and their ducts opened into the urethra, along with the vafa deferentia, at the ufual place. The internal furface of the urethra was ftudded with pellicles, as is ufually the cafe; the penis too, and the parts immediately connected with it, appeared every way compleat and perfect. The urinary bladder was connected to the uterus by cellular membranes and its peritonæal covering; it had no connection with the vaginal pouch, but was joined to the penis in the common way.

It

It has been an opinion received amongſt many phyſiologiſts, that when an animal of a perfect order* is brought forth an hermaphrodite, that it muſt have been the conſequence of a double impregnation, and that ſuch a production will be incapable of propagating its ſpecies.

With reſpect to the birth of the animal above deſcribed, no information could be procured, nor can we be more certain with regard to its fecundating powers; but if we may be allowed to form ſome judgment from the exact reſemblance of its male organs (in every eſſential particular) to thoſe of a perfect ram at the ſame age, then there does not ſeem to be any reaſon, from the ſtructure of the parts, why the animal, if it had grown up, ſhould not have had the natural propenſities of the ram. When the teſticles are detained in the abdomen, Mr. Hunter conſidered them as being always imperfect†, but in all thoſe caſes which came under his obſervation, both the ſize and ſtructure of the teſtes were evidently defective; but in the preſent caſe no deficiency in the ſize, or deviation from the natural ſtructure was apparent, and their cloſe connection with the Fallopian tubes, by the reflection of the peritonæum, will ſufficiently explain the reaſon why they did not deſcend into the ſcrotum. The early death of this animal is to be regretted, for had it arrived at maturity, it might have taught us whether ſuch a ſpecies of monſtroſity can ever ſhew partiality for the female, and how far it could have been able to propagate its ſpecies. The probability is, that the preſence of the uterus, imperfect as it is, would ſo far have checked or interfered with the natural propenſities of the male, that the animal would have ſhewn little or no partiality for either ſex.

### EXPLANATION OF THE DRAWING.

### FIG. 1ſt

A. A. The teſtes.

B. B. The ſpermatic veſſels, injected.

C. C. The epididymis, filled with quickſilver from the vas deferens.

D. D. The uterus.

E. E. The horns of the uterus.

F. F. The Fallopian tubes, terminating in a convoluted manner, and opening upon the teſticle on each ſide.

G. G. G. G. The vaſa deferentia, ariſing ut of the epididymis, and preſſing upon the outſide of the horns of the uterus.

H. H.

* I only allude to the bull and the ram, not having had an opportunity o seeing any account of the like monstrosity taking place in any other animal.

† Observations on certain parts of the animal economy, page 18.

H. H. The veficulæ feminales of the ufual fize, their ducts entering the caput gallinaginis, along with the vafa deferentia.

I. The vagina, terminating in a cul de fac.

K. The urinary bladder.

L. The membranous part of the urethra, encircled by a fphincter mufcle

M. One of Cooper's glands.

N. The bulb of the urethra.

O. The right crus of the penis, feparated from the pubis.

P. The penis.

Q. The glans, with a briftle introduced into the urethra.

FIG. II.

The vagina and uterus laid open, fhewing the internal rugofe appearance ufually met with in the perfect uterus.

A. A broad femi-lunar valve, fomewhat correfponding to the os tincæ.

B. B. The horns of the uterus lined by a fmooth membrane, wanting the glandular bodies conftantly found the perfect uterus.

FIG. III.

The membranous part of the uretha laid open into the bladder, fhewing briftles introduced into the occuli gallinginis; alfo two other briftles in the ureters.

## Figures and Tables

**Fig. 1. Fig. 2. Fig. 3. f1:**